# Clinical audit of dynamic mobilisation exercise prescription and standards of health records in veterinary physiotherapy practice

**DOI:** 10.18849/ve.v10i3.725

**Published:** 2025-08-29

**Authors:** Rebekah Davidson+, Cathy McGowan+, Kathryn Long+

**Keywords:** ANIMAL PHYSIOTHERAPY, CLINICAL AUDIT, CLINICAL EDUCATION, QUALITY IMPROVEMENT

## Abstract

**One-sentence summary:**

Veterinary physiotherapists did not adhere to the standards for the prescription of dynamic mobilisation exercises nor the Physiotherapy Board of New Zealand standards for health recording during the horse’s first appointment, but marked improvement was observed on re-audit.

**Abstract:**

**Background:** Dynamic mobilisation exercises (DME) are popular exercises prescribed during equine rehabilitation, however the application of these exercises in clinical practice has not been investigated. Evidence-based practice and accurate health recording are important for quality improvements in practice.

**Aims and objectives:** The aim of this study was to improve DME prescription quality and ensure documentation meets Physiotherapy Board of New Zealand governing board standards. The objectives were to establish an evidence-based standard of prescription, to audit the prescription dosage, program duration, combination of exercises, the accuracy of the veterinary physiotherapists (VP) clinical notes and the ability of the client to achieve the correct exercise technique.

**Methods:** Audit criteria were derived from a literature review. Retrospective data was collected from the health records of two VP, from the same practice and recorded on the data collection tool. Improvement was measured via a re-audit comparison.

**Results:** Initial audit found that none of the 26 cases met criteria standards. Exercises were not prescribed with the correct dosage, program duration, or in conjunction with gym exercises. Health records indicated the exercise parameters but did not record if clients could achieve the correct technique.

**Implementation of changes (team discussion & changes made):** Intervention included education for VP. Templates of health records and exercises were also implemented.

**Re-audit:** No changes to the audit criteria or method were made. The correct technique was not achieved by 17/27 (62%) of the clients involved in the re-audit. This did not meet the criterion standard. All remaining audit standards were achieved.

**Application:** The recommendations that were implemented resulted in more standardised DME prescription between the VP. Both VP became homogeneous with the dosage, program durations, exercise descriptions, and implemented accurate recording of their program parameters. Other practices should consider their own audits, educate practitioners, and implement customised templates.

## Introduction

Dynamic mobilisation exercises (DME) are frequently prescribed by VP and other equine health practitioners for treating the signs of back pain after following treatment or referral from a veterinarian (McGowan et al., 2007). Multiple studies have investigated the physiological effects of DME, concluding that in healthy horses under controlled conditions, DME increase intervertebral joint range of motion (ROM), improve posture, increase multifidus muscle group (*mm. multifidi*) cross sectional area (CSA), reduce *mm. multifidi* asymmetry, and improve stride quality (Stubbs et al., 2011; Tabor, 2015; de Oliveira et al., 2015; Shakeshaft & Tabor, 2020).

Effective DME prescription requires that VP align their exercise programs with the research. To measure whether evidence-based practice is being applied, a clinical audit can be used. A clinical audit measures a specific process or outcome against standards developed from evidence-based medicine; to identify areas of patient care requiring improvement and ensure good medical practice (Esposito & Dal Canton, 2014; Paton et al., 2015). Clinical audits measure current practice and may improve the patient experience of care (Paton et al., 2015). In the absence of published clinical guidelines for DME prescription in horses, best practice recommendations can be derived from the highest quality research to set criteria for audit standards; this is a criterion-based audit (UH Bristol Clinical Audit Team, 2017).

### Background

Back pain is responsible for poor performance in many horses and has a strong association with the appearance of lameness which may present as altered gait parameters or postural changes (Landman et al., 2004; Wennerstrand et al., 2009; Tabor, 2022). Reduced stride length, diminished thoracolumbar flexion/extension, spontaneous changes in pace, poor hindlimb impulsion, changing tracks, reduced lateral flexion, a lordotic static posture, and altered ridden posture are all well documented signs of equine back pain (Wennerstrand et al*.*, 2004, 2009; Mayaki et al., 2020; Dyson & Pollard, 2020; Shakeshaft & Tabor, 2020; Tabor, 2022).

Poor* mm. multifidi* motor control increased fatty infiltration and reduced cross sectional area of *mm. multifidi* has been documented in humans with chronic non-specific low back pain (Abdelaty et al., 2024). In humans, the inhibition and subsequent atrophy of *mm. multifidi* is seen as early as 24 hours following injury and causes an increased neutral zone on the ipsilateral vertebral segment; increasing the risk of recurrent injury, osteoarthritis, and other pathologies (Panjabi, 1992; Hides et al., 1996; Indahl et al., 1997; Danneels et al., 2001). Research in humans has shown that isometric contractions reverse cortical neuromuscular inhibition, which reduces pain by directly improving motor function and proprioception (Fryer, 2011; Rio et al., 2015). Increased *mm. multifidi* strength and improved local segmental motor function because of isometric contractions, increases segmental stability and contributes to the resolution of the signs of back pain (Richardson & Jull, 1995; O'Sullivan et al., 1997). Physiotherapists typically use graded exercise programs in both humans and horses that target the elimination of compensatory strategies to reverse *mm. multifidi* inhibition (Stubbs & Clayton, 2008; McLean et al., 2013; Fernández-Rodríguez et al., 2022).

In horses, DME are the isometric exercises commonly used to target the reversal of *mm. multifidi* inhibition (Stubbs & Clayton, 2008). Equine studies investigating the effects of DME programs showed that DME improved *mm. multifidi* symmetry and increased *mm. multifidi* CSA following programs of various durations (Stubbs et al., 2011; Tabor, 2015; de Oliveira et al., 2015; Lucas et al., 2022). The restoration of symmetry may represent a reversal of the inhibitory pathways and removal of compensatory strategies, whilst increased *mm. multifidi* CSA may indicate improved strength and motor function (Stubbs et al., 2011; Tabor, 2015; de Oliveira et al., 2015; Lucas et al., 2022).

Dynamic mobilisation exercises are baited, unmounted exercises which are performed by the horse standing square, on level ground (Stubbs & Clayton, 2008). The three movements used in the research are cervical flexion, lateral cervical flexion, and cervical extension (Stubbs et al., 2011; Tabor, 2015; de Oliveira et al., 2015; Shakeshaft & Tabor, 2020). When combined with gym exercises, DME improve stride quality measured via video analysis in horses (de Oliveira et al., 2015). Pelvic tilt, backing up, tight circles, and pole work are gym exercises that assist in increasing stride length and tracking distance in unridden horses (de Oliveira et al., 2015). Furthermore, following one repetition of DME static thoracolumbar posture presented with increased flexion which was maintained for an hour following completion (Shakeshaft & Tabor, 2020).

The research studies to date have all used the same exercise program; three cervical flexion exercises (chin-to-fetlock, chin-to-carpus, then chin-to-chest), three lateral cervical flexion exercises (chin-to-girth, chin-to-hock, then chin-to fetlock bilaterally) and one cervical extension exercise (Stubbs et al., 2011; Tabor, 2015; de Oliveira et al., 2015; Shakeshaft & Tabor, 2020; Gómez et al., 2022). Each of the 10 exercises was held for five seconds, and the complete set repeated five times, three times a week (Stubbs et al., 2011; Tabor, 2015; de Oliveira et al., 2015; Shakeshaft & Tabor, 2020; Lucas et al., 2022). The most significant changes in *mm. multifidi* CSA were seen in the initial six weeks (Tabor, 2015).

Furthermore, annotation of DME in the health records of horses should accurately reflect prescription, to ensure compliance with the Physiotherapy Board of New Zealand health record standard regulations regarding the health records of humans (Physiotherapy Board of New Zealand, 2018). Of relevance, physiotherapists who treat humans are required to accurately document any information given to the client and describe all treatments and interventions that occurred in the treatment session (Physiotherapy Board of New Zealand, 2018). Maintenance of these standards is required for ongoing registration as a qualified physiotherapist treating humans and animals. Previous audits of physiotherapy health records regarding the treatment of humans have shown a history of inadequate health recording within the profession and lack of compliance to the standards (Phillips et al, 2006; Cochrane, 2019; Gumery et al., 2000).

### Aims and objectives

The primary aim of this audit was to improve the quality of DME prescriptions, by two VP from the same clinic, to ensure that exercises were being prescribed in accordance with evidence-based practice. The secondary aim was to ensure that documentation of these exercises meets the Physiotherapy Board of New Zealand governing board standards. The primary objectives were to determine if the current prescription meets the audit criteria for exercise dosage, program duration, and combination of exercises. The secondary objective was to ensure that the correct exercise technique was taught to and achieved by the client in the initial appointment.

## Methods

### Audit criteria

The six audit criteria and the evidence from which they were derived are displayed in Table 1. A literature review, conducted on PubMed, Wiley Online Library, and Google Scholar provided the evidence that informed the audit criteria. The search terms included ‘dynamic mobilisation exercise’, ‘baited activation’, and ‘horse’ or ‘equine’. One abstract and seven articles were identified. Articles were published between 2010 and 2022. The evidence level was a combination of Level 2 and Level 3, graded out of 5 using the Oxford Centre of Evidence Based Medicine 2011 Level of Evidence (OCEBM Levels of Evidence Working Group, 2011). The studies are a combination of cohort studies and randomised controlled trials.

**Table 1:** Six audit criteria and their targets used to define compliance to evidence-based practice.

Criteria	Audit target	Exception	Evidence
Exercises are initially prescribed with a dosage of 5 sets, 5 second duration, 3 times a week.	90%	None.	(Stubbs et al*.*, 2011; de Oliveira et al*.*, 2015)
Exercises are initially prescribed with a repetition dosage of: 3x cervical flexions, 1x cervical extension, 3x lateral flexions bilaterally.	100%	None.	(Stubbs et al., 2011; Tabor, 2015)
Initial exercise programs for DME are 6 weeks.	90%	None.	(Tabor, 2015)
DME are prescribed in conjunction with gym exercises (pelvic tilt, backing up, tight circles, pole work).	80%	Gym exercises are contraindicated in the case and there is evidence of this in the clinical notes.	(de Oliveira et al., 2015)
Health records indicate the prescribed direction, number of repetitions, duration, repeatable explanation of procedure, required repetitions per week.	100%	None.	(McDowell, et al., 2014; Physiotherapy Board of New Zealand, 2018)
The correct technique is achieved by the client.	100%	None.	(Stubbs & Clayton, 2008)

### Audit method

The initial audit was undertaken in June 2022 by one of the VP partaking in the audit. Electronic health records were collected retrospectively from both VP belonging to a single practice. Retrospective document data was used to mitigate the Hawthorne effect, whereby people adjust their behaviour because they are aware of being observed (McCarney et al., 2007). Both VP consented to participate in the audit. The sample size was determined by the number of cases seen. All cases seen in February, March, and April 2022 were used with the timescale determined by the resources available. Cases were included in the audit if DME were prescribed for the first time. Initially any of the terminology, carrot stretches, baited activations, baited stretches, or DME when naming the exercises was included in the audit. Cases were excluded if DME were not prescribed, or DME were being progressed as part of a follow up appointment.

Clinical health records were assigned unique identifying codes and copied into a separate folder to preserve the original health records. The clinical health records were assessed for compliance to the six audit criteria via the data collection tool (see supplementary material 1). The data collection tool was translated to a spreadsheet to make analysis of the results simple. Criteria that were not present in the clinical health records scored 0 and criterion that was present scored 1. Data analysis of the outcomes highlighted criteria that did not meet the audit standards. The results informed the implementation of a new service designed to improve the quality of the DME prescription and improve consistency between the two VP. A prospective re-audit followed in February, March, and April 2023 to assess the effect of these recommendations on the quality of the DME prescription.

### Data analysis

Each criterion on the data collection tool was analysed separately to assess where intervention was required. A percentage of cases to achieve the criteria was calculated by:

(number of cases to achieve criteria ÷ total number of cases) x 100.

Scores that did not meet the predetermined audit standard were flagged for improvements. This was assessed on an achieved or did not achieve basis; no range of acceptable outcomes was applied as the criteria were either present in the clinical health records, or they were not.

### Discussion of data and plan for implementing change

At the conclusion of the initial audit cycle, the results were presented to the VP involved. Outcomes and potential resolutions were discussed to ensure both VP agreed with the implementable and realistic changes. Requirements for effective health records and exercise templates were discussed amongst the VP. An email summarising the changes required followed this meeting to ensure both VP were informed of the necessary changes to practise.

### Re-audit plan

Re-audit occurred during the same months, the following year. This was to ensure a similar cohort of horses due to the weather conditions and competition level during these three months. The method of data collection and analysis did not change, but the notes were collected and analysed prospectively.

## Results

### Initial audit findings

The initial audit comprised 26 health records from the audit period. Table 2 summarises the results of both audit cycles. The initial audit revealed that the exercise dosage, repetitions, program duration, prescription of gym exercises, and the capability of clients to achieve correct technique in the initial appointment did not meet target standards. Clinical health records indicated the exercise parameters every time. Fifty-eight percent of clients received the correct dosage, 0% received the correct repetitions, 27% of clients were informed of the program being six weeks long, 77% of clients received gym exercises with their DME, and 0% of the clinical notes reported that clients were shown the exercises and can complete them. 100% of health records appropriately indicated direction, number of repetitions, hold time, instructions, and number of sets per week.

**Table 2:** Initial audit and re-audit findings representing the compliance of the clinical health records to the 6 audit criteria for a total of 53 cases from an equine physiotherapy practice in New Zealand.

	Initial Audit (n = 26)		Re-Audit (n = 27)		
Criteria	Cases achieved to target standard	% Achieved to target standard	Cases achieved to target standard	% Achieved to target standard	Audit Target
Exercises are initially prescribed with a dosage of 5 sets, 5 second duration, 3 times a week on the initial prescription.	15	58%	27	100%	90%
Exercises are initially prescribed with a repetition dosage of: 3x cervical flexion, 1x cervical extension, 3x lateral flexion bilaterally.	0	0%	27	100%	100%
Exercise programs for DME are 6 weeks.	7	27%	26	96%	90%
DME are prescribed in conjunction with gym exercises (pelvic tilt, backing up, tight circles, pole work).	20	77%	22	81%	80%
The correct technique was taught to and achieved by the client in the initial appointment, which is annotated in the health record.	0	0%	17	62%	100%
Health records indicate the prescribed: direction,	26	100%	27	100%	100%
number of repetitions,	26	100%	27	100%	100%
amount of time held,	26	100%	27	100%	100%
repeatable explanation of procedure,	26	100%	27	100%	100%
required repetitions per week.	26	100%	27	100%	100%

Incidental findings that were not directly measured by the initial audit included clinical health records that did not accurately reflect the physiotherapy session. Veterinary physiotherapists self reported teaching the correct DME technique to clients but there was no evidence of teaching the correct DME technique to clients in the health records. Written exercise descriptions were difficult to interpret without pictures or clear instructions, nor did the instructions obviously state the prescribed number of repetitions and sets. Exercise dosage varied between VP, reflecting an inconsistency in the interpretation of the research and differences in the accuracy of health recording. Furthermore, exercises were often separated in an attempt to target specific areas of the spine, but DME movements have not been investigated singularly for their effect. Currently, there is no evidence to support the effectiveness of the exercises in isolation, nor is there evidence to support a dosage that differs from those used in this audit.

## Implementation of changes (team discussion & changes made)

Recommendations for quality improvement included training VP in the correct technique used for DME and gym exercises. This included education regarding the effects of DME, the recommended dosage, program duration, and the recognition of appropriate case selection based on the evidence. The accuracy of clinical health records and streamlining the health recording process was improved by supplying the VP with comprehensive health record templates (see supplementary material 2). Exercise description templates (see supplementary material 3) were also provided with photos, clear instructions, and obvious prescription of repetitions and sets that aligned with the audit criteria. The intervention plan to achieve these recommendations is summarised in Table 3. Both physiotherapists committed to the re-audit cycle.

**Table 3:** The intervention plan that was used to improve adherence to audit standards and the governing board health record standards.

Action	Responsible VP	Start date	End date
Present the audit findings to the VP involved.	VP1	6/6/22	6/6/22
Discuss recommendations and agree on realistic changes to practise.	VP2 VP1	6/6/22	6/6/22
Consolidate DME techniques to ensure VP prescribe them in the same format and technique.	VP1	7/6/22	8/8/22
Consolidate gym exercise techniques to ensure VP prescribe them in the same format and technique.	VP1	7/6/22	8/8/22
Present the correct method, technique and format to the other VP and train VP accordingly.	VP1	22/8/22	22/8/22
Write detailed exercise templates.	VP1	7/6/22	22/8/22
Write detailed health record templates.	VP1	7/6/22	22/8/22
Share templates with VP.	VP1	22/8/22	22/8/22
Train all VP in the correct use of the templates.	VP2 VP1	22/8/22	22/8/22
Email bullet points to summarise the required changes for VP.	VP1	25/8/22	25/8/22
Present the audit findings to the VP involved.	VP1	6/6/22	6/6/22
Discuss recommendations and agree on realistic changes to practise.	VP2 VP1	6/6/22	6/6/22

Barriers to change included the increased time required for recording exercises in the recommended format and the introduction of new technologies to do so. This was mitigated by comprehensive training, so VP understood the effects of DME, highlighting their importance in the rehabilitation of horses with back pain. Training was provided in person and included the findings of the literature review, the effects of the exercises and practice on live horses to ensure correct technique and mitigation of compensatory strategies. Veterinary physiotherapists discussed appropriate case selection based on the literature review regarding changes in posture and gait parameters (Landman et al., 2004; Wennerstrand et al., 2009; Tabor, 2022). Both VP left the training confident in their prescriptions of DME. Exercise and health record templates were placed in a shared folder on Google Drive^TM^ and the VP reported a reduced note completion time despite the time used to train VP in the new technology.

## Re-audit results

The re-audit included 27 cases, different from those that received their initial prescription of DME in the initial audit; results are summarised in Table 2. The audit criteria for the correct technique was being taught and achieved by the client in the initial appointment was not met, with only 62% of cases meeting the standard. Failure to meet the audit target of 100% target reflects a second update in the clinical health record template where this criterion (the correct technique was being taught and achieved by the client) was omitted from the template. Veterinary physiotherapists report still teaching their clients the exercises during the time, but as it was not recorded in the clinical notes, it could not be registered as achieved. To resolve the omission, the template was reworked for a third time and will continue to be developed to meet the changing requirements of the clinic.

## Discussion

This audit was undertaken to improve the quality of DME prescription and recording in the health records. It was expected that there would not need to be any changes to clinical practice as the VP believed they were already delivering a high standard of health care, however, this was not achieved until the re-audit a year later. An audit allows insight into how current practice measures against industry standards and performance (Rose & Pang, 2021). Therefore, the audit cycle reported here allowed the involved VP to understand the baseline at which they were operating and highlight where improvements were needed. The high rate at which audit targets were met during re-audit highlights that it is possible to meet the targets and provide quality DME prescriptions to horses with back pain. Furthermore, it appeared important to have a comprehensive understanding of the available literature when aiming to achieve and maintain high standards of prescription. The participating VP were not able to achieve the audit targets until they had undergone thorough training in the effects and application of DME. It appears that regular review of the most recent literature is required to ensure that physiotherapists “use evidence-based practice to support clinical decision-making” (Physiotherapy Board of New Zealand, 2023).

There is currently a lack of comparable studies investigating the application of DME by VP in clinical practice. However, Waine et al. (2021) indicated that retrospective data collection within the large animal veterinary sector has been difficult due to the poor standard of health records. This audit cycle found that to be true. Particularly regarding the ability of the client to achieve the correct technique in the initial appointment. This criterion was cause for confusion as it was unclear whether the audit targets were not met due to clients not being taught the correct technique, not being able to complete the correct technique or this simply not being recorded. It would be of value to investigate these terms separately.

Published guidelines regarding DME prescription parameters were unavailable at the time of the audit, meaning the audit criteria and targets were derived from multiple research articles as shown in Table 1. Firstly, research in this area is limited and more research into the individual movements is required to understand the effects of DME properly. Secondly, multiple sources caused confusion as to what was being measured. Repetitions, sets, and duration are all forms of dosing an exercise, but these were split up on the assessment tool into two separate and unnecessary criteria. As more research into the effects of DME is undertaken, the audit criteria should be modified for re-audit, to ensure that VP are maintaining evidence-based practice to support “clinical  decision-making” (Physiotherapy Board of New Zealand, 2023). Furthermore, during the re-audit, VP participants were aware of the initial audit results and aware of their data being audited again, so the results may have been biased to be more positive (Esposito & Dal Canton, 2014). To mitigate this, the physiotherapists should have had the re-audit undertaken by an external auditor, without their awareness. The strict time frame for the audit and re-audit cycle meant that there were a limited number of cases included in the audit. The number of cases included was restricted by the number of clients the VP were able to see within the three months of the audit. Potentially, the number of cases may not be sufficient to accurately represent the entire case load.

Incidental findings of this audit cycle indicated that there should have been more audit criteria dedicated to the clarity of clinical health records and exercise description handouts. These findings imply that simply following evidence-based practice is not enough to provide high quality health care. Consideration of the client experience and in this case the equine owners, is also important.

## Conclusion

This audit provides an overview of the current practise of prescribing DME in a veterinary physiotherapy practice in New Zealand. The findings of the first audit cycle indicated that DME prescription did not meet the targets for the audit criteria derived from the research. Lack of awareness by the participating VP surrounding the research recommendations was the primary reason for poor performance in the initial audit highlighting the need for audit in clinical practice. The criteria derived from the research provides a proven and achievable benchmark for evidence-based practice in VP practice. The intervention implemented was successful in allowing the re-audit criteria to be met, improving physiotherapist compliance to the recommendations and standards of health records. Further audit cycles in this veterinary physiotherapy practice should align with more recent research and should consider the discrepancies in educating the client correctly versus accurately recording the DME prescription.

## Application

This audit was fundamental in measuring the prescription of DME in veterinary physiotherapy. Clinical audit was found to be an effective quality improvement tool and is recommended for other practices. However, it is unknown whether the inadequacies identified by this audit also occur in other practices. Furthermore, other practices will encounter unique barriers to applying research findings. The intervention used in this audit cycle standardised the prescription of DME within the practice but it is acknowledged that other practices may require customised improvement plans to achieve similar results.

## Ethical approval

This project was exempt from ethics approval because it was an audit of current clinical practice. No measures were being taken and no intervention applied to any animals and animal owners were not involved. Therefore, the New Zealand Ministry for Primary Industries does not require ethical approval.

## Informed consent

Informed consent was obtained from the Veterinary Physiotherapists who participated in this audit (and are both authors RD and KL).

## Supplementary materials

Supplementary material S1 – Supplementary 1: Data Collection Tool.

Supplementary material S2 – Supplementary 2: Health Record Template.

Supplementary material S3 – Supplementary 3: Exercise Description Templates.

## Author contributions

**Rebekah Davidson:** Conceptualization, Methodology (lead), Investigation, Formal Analysis, Writing- Original draft preparation. **Cathy McGowan:** Supervision,  Conceptualization, Methodology (supporting), Reviewing and Editing (supporting). **Kathryn Long:** Investigation, Writing- Reviewing and Editing (supporting).

## ORCiD

Rebekah Davidson: https://orcid.org/0009-0008-3751-9136

Cathy McGowan: https://orcid.org/0000-0002-1946-9584

Kathryn Long: https://orcid.org/0000-0002-6275-6386

## Conflict of Interest

The authors declare no conflicts of interest.
